# Impact of Extraction Method on the Structure of Lignin from Ball-Milled Hardwood

**DOI:** 10.1021/acssuschemeng.3c02977

**Published:** 2023-10-19

**Authors:** Ioanna Sapouna, Gijs van Erven, Emelie Heidling, Martin Lawoko, Lauren Sara McKee

**Affiliations:** † Wallenberg Wood Science Center, KTH Royal Institute of Technology, 114 28 Stockholm, Sweden; ‡ Division of Glycoscience, Department of Chemistry, KTH Royal Institute of Technology, AlbaNova University Center, 114 21 Stockholm, Sweden; § Wageningen Food and Biobased Research, Wageningen University & Research, Bornse Weilanden 9, 6708 WG Wageningen, The Netherlands; ∥ Laboratory of Food Chemistry, Wageningen University & Research, Bornse Weilanden 9, 6708 WG Wageningen, The Netherlands; ⊥ Division of Wood Chemistry and Pulp Technology, Department of Fiber and Polymer Technology, KTH Royal Institute of Technology, 114 28 Stockholm, Sweden

**Keywords:** biomass, lignin characterization, nuclear magnetic resonance spectroscopy, Py-GC-MS, solvent fractionation

## Abstract

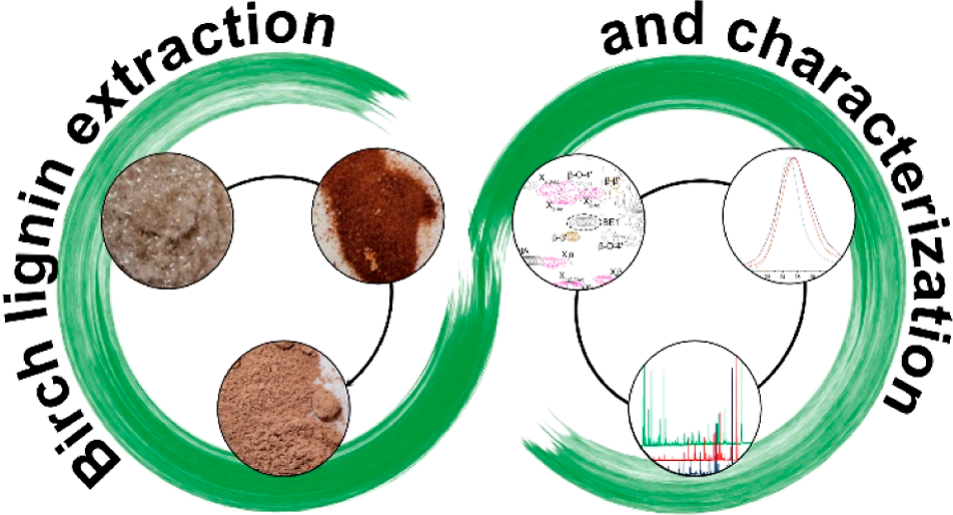

Understanding the structure of hardwoods can permit better valorization of lignin by enabling the optimization of green, high-yield extraction protocols that preserve the structure of wood biopolymers. To that end, a mild protocol was applied for the extraction of lignin from ball-milled birch. This made it possible to understand the differences in the extractability of lignin in each extraction step. The fractions were extensively characterized using 1D and 2D nuclear magnetic resonance spectroscopy, size exclusion chromatography, and pyrolysis–gas chromatography–mass spectrometry. This comprehensive characterization highlighted that lignin populations extracted by warm water, alkali, and ionic liquid/ethanol diverged in structural features including subunit composition, interunit linkage content, and the abundance of oxidized moieties. Moreover, ether- and ester-type lignin–carbohydrate complexes were identified in the different extracts. Irrespective of whether natively present in the wood or artificially formed during extraction, these complexes play an important role in the extractability of lignin from ball-milled hardwood. Our results contribute to the further improvement of lignin extraction strategies, for both understanding lignin as present in the lignocellulosic matrix and for dedicated lignin valorization efforts.

## Abstract 2

Systematic characterization of hardwood lignin obtained via green protocols reveals useful lignin populations that can be targeted for extraction.

## Introduction

For society to move toward a sustainable future, technologies that enable the effective valorization of biomass to produce materials that can replace fossil-based analogues are needed. Wood biomass is used for a plethora of products, from the most common pulp and paper, to inks,^1,2^ bioplastics,^3^ films,^4^ biofuels,^5,6^ and more.^7,8^ The structure of native lignin and its interactions with the polysaccharides of the plant cell wall have been a point of controversy for many years.^9,10^ Consequently, this abundant biopolymer is still underutilized, mainly serving as a fuel for pulp mills, through combustion.^7^ Significant efforts have been made toward higher-value lignin utilization, mostly focusing on materials made from Kraft lignin, the most common so-called technical lignin derived from black liquor generated in the pulping process.^7^ However, in most cases, Kraft lignin needs to be modified after extraction to obtain the necessary properties. This is a tedious process due to the complexity and heterogeneity of its structure. In this work, we promote the use of mild extraction protocols that preserve the structural characteristics of lignin, which can then be used without further modification.

To this end, we previously developed a green protocol for the extraction of lignin from ball-milled softwood and studied the impact of extraction on the structure of lignin.^11^ However, the lignin composition and structure exhibit fundamental differences between softwood and hardwood species. In general, the three main building blocks of lignin are guaiacyl (G-), syringyl (S-), and small amounts of *p*-hydroxyphenyl (H-) units, respectively, derived from coniferyl, sinapyl, and *p*-coumaryl alcohol precursors.^10^ Softwood lignin is almost completely composed of G-units, whereas hardwood lignin has a mixture of G- and S-units, the latter of which are generally more abundant.^10^ Besides lignin, softwoods and hardwoods also differ in hemicellulose composition, in terms of the substitution pattern, degree of acetylation on glucuronoxylan, and an enrichment of (galacto)glucomannans in softwood, leading to different types of lignin–carbohydrate complexes (LCCs) as a result.^12^ These features affect lignin’s extractability in different processes, so it is important to understand the structure and extraction behavior of lignin in both wood types to be able to target this abundant polymer for extraction.

A “lignin-first biorefinery” is a concept that has gained attention lately and focuses on the in situ depolymerization of lignin.^13^ The concept can also include mild methods that directly target lignin moieties with desired properties for extraction from biomass, without the need to first completely degrade the wood.^14^ Toward this goal, understanding the effect of extraction on the structure of lignin is of utmost importance. Recent studies on biomass extraction with ionic liquids,^15−17^ enzymatic treatments,^18^ or solvent systems^18−22^ focus on polysaccharide valorization, and there has been rather limited characterization of the extracted lignin structure. To the best of our knowledge, the extraction methods applied to hardwood biomass have not yet been studied in depth in terms of the structural moieties they can extract, so the value of extracted lignin for applications remains unclear.

This study aims to address the effect of mechanical pretreatment and different extraction protocols on the structure of hardwood lignin. Our focus is to understand the differences in lignin extractability in different process steps, and for that purpose, the structure of several lignin extracts has been characterized using nuclear magnetic resonance (NMR) spectroscopic techniques [heteronuclear single quantum coherence (HSQC) and phosphorus-31 NMR (^31^P NMR)] and size exclusion chromatography (SEC). In addition, a comprehensive evaluation of the process was achieved using pyrolysis–gas chromatography–mass spectrometry (Py-GC-MS) analysis, allowing the characterization of both the lignin extracts and insoluble wood residues. The importance of selecting the appropriate protocol for lignin extraction is shown in this work. Specific chemical properties are sometimes required for different material applications, and this study highlights how those needs can be met by designing the wood extraction strategy accordingly. If it is possible to target the desired properties by the extraction protocols alone, without the need for modification of the extracts, it is possible to develop greener and more sustainable extraction protocols.

## Experimental Section

### Chemicals and Materials Utilized

All the chemicals were purchased from Sigma-Aldrich and used without purification unless specified. Absolute ethanol was purchased from VWR, Sweden.

### Mechanical Pretreatment of Biomass

Two milling steps were sequentially applied to the wood for efficient particle size reduction.

### Wiley Mill

Debarked birch wood chips were milled with a Wiley mini mill (Thomas Scientific, USA) through a 20-mesh sieve. Spruce wood chips used for scanning electron microscopy (SEM) analysis were screened through a 40-mesh sieve.

### Planetary Ball Mill

The particle size of the wood meal was further reduced using a planetary ball mill (PM400, Retsch, Ninolab, Sweden). The process was adapted from that described in a previous publication.^11^ In short, stainless steel grinding jars (500 and 250 mL) were used with 1 cm diameter stainless steel grinding balls, with a jar volume (L)/grinding balls (kg)/sample weight (g) ratio of 1:0.8:40. Ball milling was performed without temperature regulation/recording at 300 rpm. The samples were milled under normal (air) and nitrogen (N_2_) atmospheres. For milling under a N_2_ atmosphere, the grinding jars were purged with the gas for 1 min prior to milling. Samples were milled following a “1 h milling—30 min break” interval pattern. The selected milling intervals were 1, 2, 12, and 24 h. The samples are named A01, A02, A12, and A24 for milling under air or N01, N02, N12, and N24 for milling under a N_2_ atmosphere, where the number describes the total milling time (h).

### Scanning Electron Microscopy

The morphology of the ball-milled birch and spruce wood particles was studied with a tabletop scanning electron microscope (Hitachi TM-1000 Tabletop SEM, Tokyo, Japan). The acceleration voltage was 15 kV, and the current was between 31.7 and 34.9 μA. 100× and 500× magnifications were used, and for all samples, the working distance varied between 5760 and 6160 μm. The samples were placed on a carbon tape for imaging and were not coated.

### Klason Lignin Determination

Sulfuric acid (H_2_SO_4_) hydrolysis was performed for the gravimetric determination of Klason lignin content as described in a previous publication.^11^ Briefly, 1 g of Wiley-milled birch was extracted with 88 mL of acetone in a Soxhlet extractor for 6 h. 200 mg of extractive-free wood meal was mixed with 3 mL of 72% H_2_SO_4_ and placed in a vacuum desiccator for 80 min with occasional stirring. 84 mL of Milli-Q water was added to the samples, which were then autoclaved for 1 h at 125 °C. The hydrolysate was filtered using a glass microfiber filter (Whatman 1820-125) and was washed twice with boiling Milli-Q water. The filter was dried overnight at 105 °C for the gravimetric determination of Klason lignin.

### Warm Water Extraction

Warm water extraction of ball-milled wood was performed according to our previous work.^11^ 10% (w/v) dispersion of ball-milled wood in Milli-Q water was stirred at 80 °C in an oil bath for 4 h. The warm water extract (denoted WWE) was collected by centrifugation for 10 min, without temperature regulation, at 4000 rpm (ROTOFIX 32 A centrifuge, rotor 1624, Hettich), and freeze-dried.

### Alkaline Extraction

Alkaline extraction was performed as in our previous publication.^11^ In short, a 10% (w/v) dispersion of the still wet wood residue from the previous extraction step was prepared in 0.1 M sodium hydroxide (NaOH), and the mixture was stirred at room temperature for 3 h. The extract was collected by centrifugation as described for the warm water extraction, and the residue was washed with Milli-Q water until pH 6 was reached. The wash liquid was pooled, together with the extract. The pH of the pooled lignin solution (wash liquid and extract) was adjusted to 2 by addition of hydrochloric acid (HCl), and the precipitated lignin was collected by centrifugation as described above, washed twice with Milli-Q water, the pH of which was set to 2 with HCl, and freeze-dried.

### Ionic Liquid Treatment

Two separate treatments with 1-allyl-3-methylimidazolium chloride ([amim]Cl) were performed on the washed, wet wood residue either after the alkaline extraction or directly following the warm water extraction.

The process followed was largely as described in previous work.^11^ In short, the wet wood residue was mixed with [amim]Cl in a 1:1 weight ratio at 80 °C for 2 h. Afterward, 80% (v/v) ethanol, also containing 0.1 M HCl in some experiments, was added to the mixture to form a 5% (w/v) dispersion. The vials were sealed, and the dispersion was stirred at 100 °C in an oil bath for 2 h. After cooling down to room temperature, the extract was collected by centrifugation as described in the previous section, and ethanol was evaporated under reduced pressure while keeping the volume constant by adding Milli-Q water. The precipitated lignin was collected by centrifugation, washed twice with Milli-Q water (pH 4 with HCl), and freeze-dried. The samples are denoted as IL/EtOH, H^+^ when HCl was included for the extraction and IL/EtOH when no HCl was added to the solvent.

### Size Exclusion Chromatography

The molecular weight and dispersity (*D̵*) of the samples were characterized by SEC. The hemicellulose-rich extracts from the WWE were analyzed using dimethyl sulfoxide (DMSO)–SEC (eluent DMSO containing 0.5 wt % LiBr), using pullulan standards for the calibration. All lignin-rich extracts were analyzed with tetrahydrofuran (THF)–SEC after acetylation, and polystyrene standards were used for the calibration. Both analytical methods are described in detail in previous work.^11^

### NMR Spectroscopy

HSQC NMR experiments were performed for the structural characterization and semi-quantification of hemicelluloses and lignin in the extracts. A Bruker 400 MHz DMX instrument (Bruker Corporation, Billerica, MA, USA) equipped with a multinuclear inverse Z-grad probe was used. The pulse sequence was hsqcetgpsi. The pulse length was optimized at 9.2 s with a 1.49 s relaxation delay and 176 scans per sample. For each experiment, approximately 70 mg of sample was dissolved in 550 μL of DMSO-*d*_6_. Phase correction in the spectra was performed manually, and baseline correction was performed using a third-order Bernstein polynomial fit. Peak assignment was made according to previous work.^11,23,24^ Calculations on the quantification of interunit linkages are provided as Supporting Information.

^31^P NMR experiments were conducted according to previous work,^25^ following the protocol by Argyropoulos.^26^ Peak assignments were made according to the study by Pu et al.^27^ A Bruker Avance III HD 400 MHz instrument with a BBFO probe equipped with a Z-gradient coil was used. The pulse sequence zgig30 was used, with 256 scans and a relaxation delay (D1) of 6 s. The spectra were processed with MestreNova (Mestrelab Research).

### Py-GC-MS with Uniformly ^13^C-Labeled Lignin as Internal Standard

To structurally characterize lignin in the extracts and residues, the samples were analyzed by Py-GC-MS).^28,29^ Analytical pyrolysis coupled to GC with high-resolution (HR) mass spectrometric detection (Exactive Orbitrap, Thermo Scientific, Waltham, MA, USA) was performed as previously described, using an Agilent VF-1701 ms column (30 m × 0.25 i.d. 0.25 μm film) for chromatographic separation.^29^ Uniformly ^13^C-labeled lignin, isolated from ^13^C willow (*Salix alba*, 96 atom % ^13^C) (IsoLife BV, Wageningen, The Netherlands) was used as an internal standard (^13^C-IS). To each accurately weighed sample (80 μg) was added 10 μL of a ^13^C-IS solution (1 mg/mL ethanol/chloroform 50:50 v/v). Samples were dried prior to analysis. Pyrolysis is performed in a microfurnace oven with the temperature set at 500 °C for 1 min. All the samples were prepared and analyzed in duplicate. Lignin-derived pyrolysis products were monitored in full MS mode on the most abundant fragment per compound (both nonlabeled and uniformly ^13^C labeled) (Table S1). Pyrograms were processed by TraceFinder version 5.1 software. Relative abundances of lignin-derived pyrolysis products were calculated as described previously.^29^

## Results and Discussion

In this work, we studied the effect of different extraction techniques on the structure of hardwood lignin by applying a sequential mild extraction protocol composed of warm water, alkali, and ethanol extraction, the last of which is applied after swelling of the substrate in an ionic liquid. The mild conditions of the protocol enabled the characterization of diverse lignin fractions using NMR techniques, SEC, and Py-GC-MS. To understand the impact of each individual process step on the yield and structure of lignin that could be extracted, modifications were applied to an initial protocol developed for spruce.^11^ The composition of the starting ball-milled hardwood, the final wood residue, and the wood residues after each individual extraction step were all investigated. This is, to our knowledge, the first systematic investigation of the impact of extraction on the structure of lignin in hardwoods.

### Ball Milling Conditions Differentially Impact Wood Particle Morphology

Reduction of particle size is an important pretreatment step used to increase the surface area of the substrate and thereby achieve higher extraction yields by increasing contact with the solvent. Ball milling is a common method applied in various protocols for biomass extraction.^30−32^ There are different studies in the literature investigating the impact of the ball milling time on the structure of lignin. For example, Wang et al. reported a reduction of β-Ο-4′ content in lignin isolated from birch, compared to that in three more biomasses.^33^ However, comparison between studies is difficult because of the different instruments used, grinding materials, and media. Nonetheless, high amounts of energy are introduced to the sample during the milling process in order to achieve sufficient particle size reduction, which leads to partial polymer degradation.^9,11,34^ Even though the temperature is regulated differently in different experimental setups, it is generally accepted that thermal energy is introduced to the substrate during ball milling.^35^ Depending on the extent of the temperature increase, some of the properties of the milled sample, for example, polymer degradation or bond formation, could be heat-induced. Our previous work on spruce showed that the ball milling atmosphere impacts the extraction yield and the intensity of milling-induced damage, qualitatively monitored through the reduction of cellulose crystallinity.^11^ In this work, the particle morphology of ball-milled birch was investigated as an indication of the ball milling intensity.

**Figure 1:**
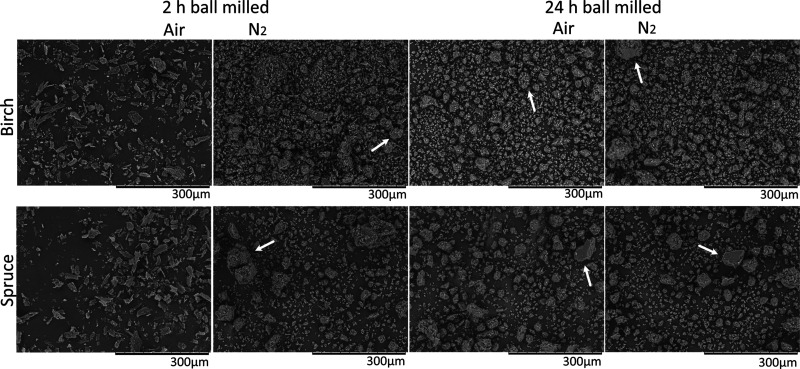
SEM images of spruce and birch samples milled under air and a N_2_ atmosphere for 2 and 24 h. Arrows are pointing to representative aggregates. The scale bar in all images is 300 μm.

The particle morphology of ball-milled spruce and birch samples explored using SEM, verified that the same degree of size reduction had been achieved for both wood types for the same milling time and atmosphere (Figure 1). It is easily observed from the comparison of samples milled for 2 h that the efficiency of the process in size reduction is higher under a N_2_ atmosphere. However, after 24 h of milling, the samples look similar, and there is no significant difference observed by SEM imaging. Because of the aggregation of the milled wood particles, especially observed for the 24 h-milled samples, as well as the heterogeneity of the shapes of the particles, the average particle size cannot be measured in the SEM images. However, as a reference, the starting birch wood meal was Wiley-milled through a 20-mesh sieve, which would correspond to 0.84 mm maximum particle size before ball milling. Accordingly, for spruce, a 40-mesh sieve was used, corresponding to 0.40 mm maximum particle size before ball milling.

Wood particle aggregation seen in Figure 1 is probably the result of the relative humidity in the atmosphere. This apparent aggregation did not seem to impact the extractability of hemicelluloses or lignin from birch, as observed by the extraction yields, explained below. However, the milling atmosphere seemed to impact not only the yield but also the abundancy of lignin interunit linkages and LCCs.

### Lignin Structures Obtained through Solvent-Mediated Extractions

**Figure 2:**
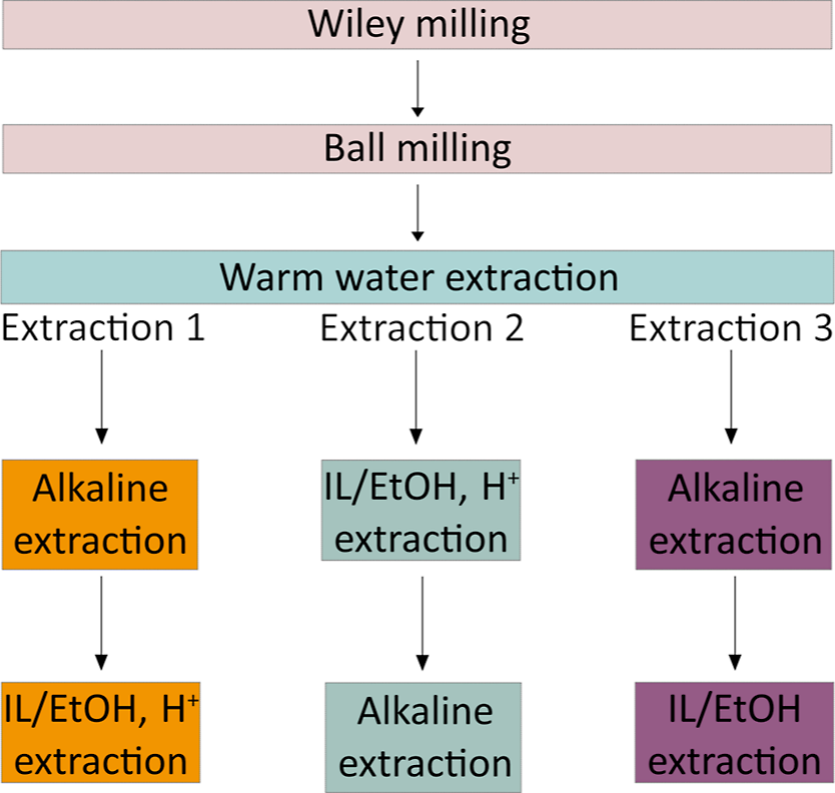
Extraction protocols developed for the study of different fractions of hemicelluloses and lignin in birch. In extraction 1, the ethanol solution containing hydrochloric acid is denoted by “IL/EtOH, H^+^”, while there is no acid in the same step of extraction 3, which is denoted as “IL/EtOH”.

A sequential extraction of hemicelluloses and lignin followed the mechanical pretreatment, as in previous work on spruce.^11^ In all steps, mild conditions were applied in order to reduce the occurrence of unwanted side reactions that would alter the native structure of lignin and to build a green fractionation approach suitable for sustainable future upscaling for dedicated valorization of functional fractions. The three extraction protocols developed are summarized in Figure 2.

**Figure 3:**
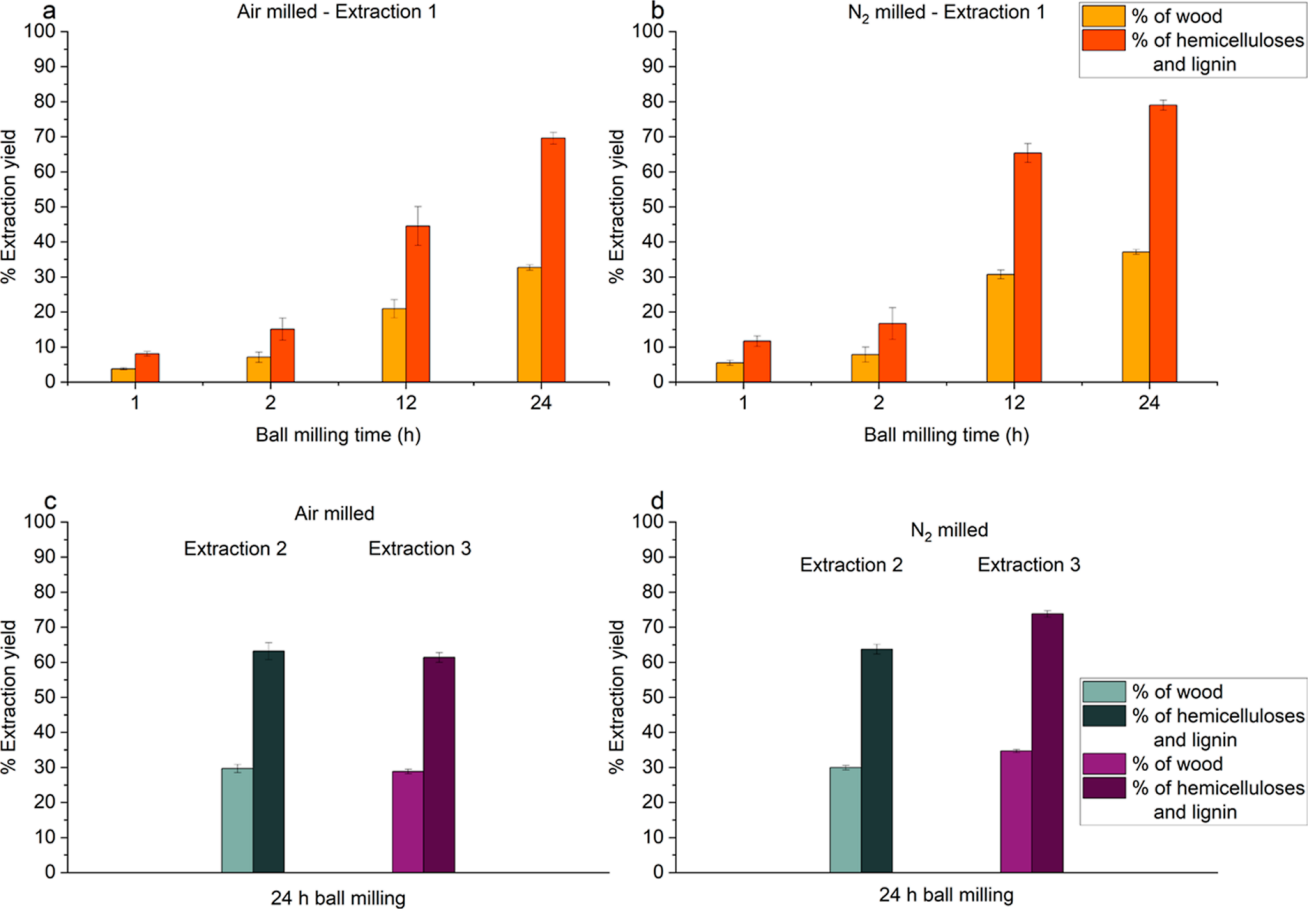
Total yields of extraction as a percentage of wood and of total hemicelluloses and lignin obtained from birch, following (a,b) extraction 1 and (c,d) extractions 2 and 3 (Figure 2). Hemicelluloses accounted for 28% based on literature,^38^ and lignin was determined gravimetrically as Klason lignin, at 19% of the wood biomass. The error bars are the standard deviation.

Hardwood lignin is enriched in S-units, and as such, it has a higher content of β-aryl ether (β-O-4′) bonds compared to softwood lignin.^10^ These ether bonds are more labile under acidic conditions and can be cleaved in certain extraction methods. Hence, a balance between high extraction yields and β-O-4′ preservation should be achieved to study representative lignin populations.^36^ Depending on the milling conditions and extraction protocol used, it was possible to obtain up to ∼79% of the hemicelluloses and lignin in the wood samples (Figure 3), which accounts for ∼37% of the hardwood biomass. As a comparison to our previous work,^11^ when the extraction 1 protocol was applied to spruce, a maximum of approximately 75% of the sample’s hemicelluloses and lignin was extracted, which accounted for ∼57% of the softwood biomass. For hardwood, the same trend of generally increased extraction yields achieved for the N_2_ milled samples was observed (Figure 3), attributed to the higher efficiency of milling. The different yields obtained when the same extraction protocol was applied to spruce and birch are an excellent way to underline the different compositions and biopolymer structural properties in softwoods and hardwoods. A slightly higher cellulose content in hardwoods, and a form of lignin that is more labile to degradation compared to that of softwoods, could account for the differences in the extraction yields.^37^ Depolymerization of lignin and hemicelluloses during extraction could lead to losses between the extraction steps because the material might end up in different fractions or might be washed away due to changed solubility and molecular weight. This should also be taken into consideration when comparing these yields.

All the extraction yields from the three protocols followed in this work are reported in Table S2 and are calculated as they were in previous work,^11^ using a combination of NMR integration and Klason lignin determination. Since HSQC is a semi-quantitative method that only accounts for the soluble part of the sample, the values are not to be treated as absolute but rather used to get a trend and relatively compare the samples. The acetone-soluble extractives were determined around 1.5 wt % during Klason lignin determination. Even though the extractives were not removed from the samples prior to sequential extraction, they would contribute only lightly to the mass balance of the fractions.

### Hemicellulose-Rich WWEs Show a Preferential Extraction of S-Rich Lignin with Longer Ball Milling Times

**Figure 4:**
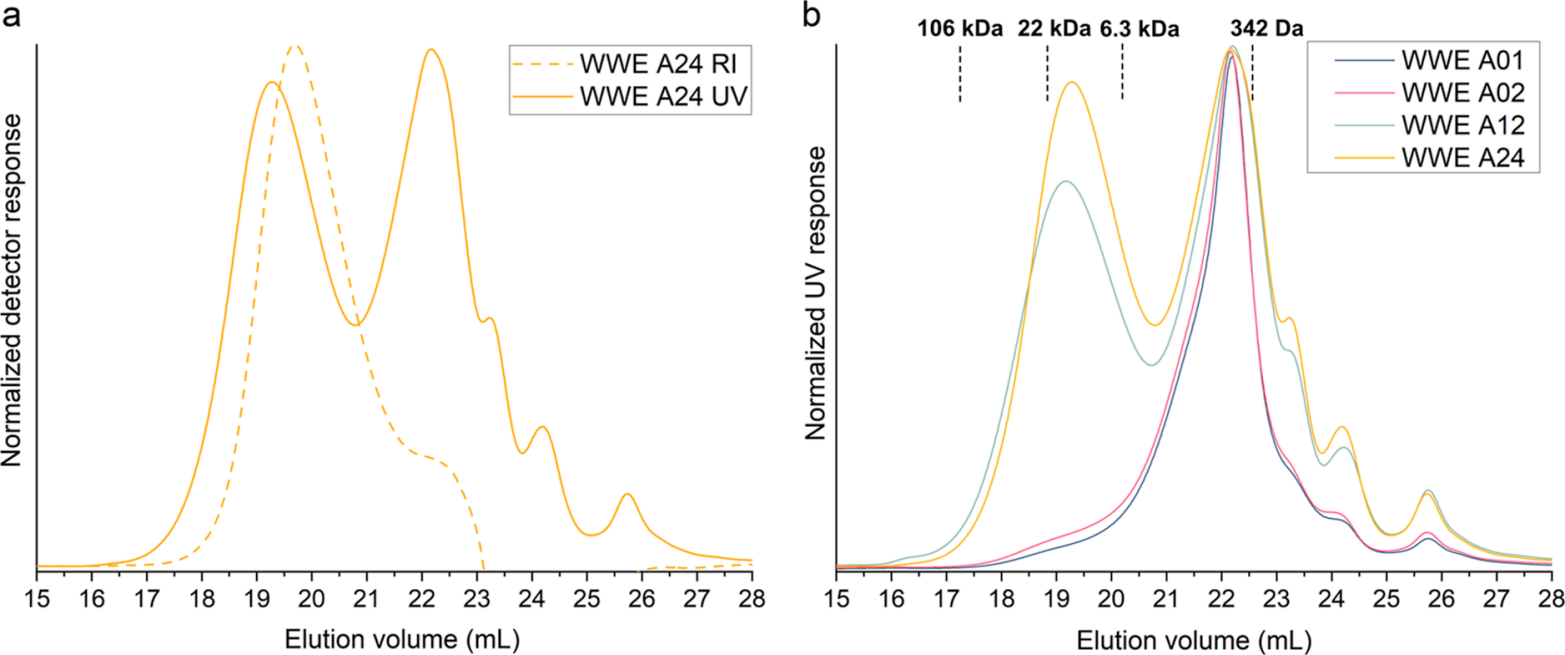
(a) Size exclusion chromatogram overlays (DMSO–SEC) of the UV and RI response for the WWE from ball-milled birch for 24 h under normal atmosphere (A24). (b) SEC overlays (UV response) of WWEs. The curves are normalized to the maximum height. All RI–UV overlay chromatograms are presented as Supporting Information (Figure S1).

The first step of the extraction, common to all routes (extractions 1–3, Figure 2), was the removal of a fraction rich in hemicelluloses soluble in hot water. Characterization by HSQC NMR showed that the amount of lignin in this fraction varied between 9.7 and 13.7%, with no specific correlation to the milling time or atmosphere (Table S2). In addition, DMSO–SEC analysis showed that the lignin moieties consisted of relatively low molecular weight oligomers that are water soluble or are solubilized by covalent attachment to carbohydrates (i.e., LCCs). The presence of the LCCs can be inferred from the overlapping signals of the RI and UV detectors (Figure 4a) as a comparison of the similar intensities of the RI (20 mL) and UV (19.5 mL) peaks and the much larger difference between the peaks at 22.5 mL suggest a coelution of lignin and hemicelluloses at 22.5 mL. The likely presence of the LCCs agrees with our previous work on spruce, where benzyl and γ-ester LCCs were identified in the equivalent fraction, even though no esters were identified in the birch WWEs.^11^

The effect of ball milling time on the lignin present in WWEs is clear in the overlay of the chromatograms (Figure 4b). With increasing milling time, a higher molecular weight lignin population emerged, which was not observed in the shorter milling intervals of 1 and 2 h. Its occurrence could mean that longer milling intervals are required for the wood structure to break down sufficiently for these populations to be extracted. On the other hand, these populations could be a result of the recombination reactions arising from the high mechanical and thermal energy introduced to the sample during milling and the formation of radicals in the sample.^39^ The recombination of smaller molecular weight moieties to form higher molecular weight moieties could be possible under these conditions.

Nonetheless, HSQC NMR analysis of the extracts milled for 12 and 24 h (Table S3) showed a similar composition in these samples. Interestingly, the β-O-4′ content is increased in the samples milled for a longer time, regardless of the milling atmosphere. A correlation between this observation and the changes in the chromatograms (Figure 4b) could be drawn; the increasing amount of β-aryl ether bonds could be a result of the larger molecular weight populations extracted with longer milling times. At the same time, the oxidized syringyl structures identified in the HSQC spectra of the WWEs at 7.24/106.1 ppm also seem to increase with longer milling times (Table S3). The same trends are again seen for both milling atmospheres, although the N_2_-milled samples in general have a higher β-O-4′ content. The other identified interunit linkages in the spectra have similar abundancies when comparing the same milling intervals for the normal and N_2_ atmospheres. Interestingly, the S/G ratio calculated from the HSQC spectra is significantly increased from 1 to 12 h milling (Table S3). It seems that after 1 h of milling, there is no preferred unit extraction with an S/G ratio of 3.0 and 2.8 for normal and N_2_ atmospheres, respectively, but after longer milling times, there is a selective S-unit extraction shown by a much higher S/G ratio of 6.4 for normal atmosphere and 7.2 for the N_2_ atmosphere (Table 2). This apparent selectivity that seems dependent on the milling time could be attributed to more G-unit-rich lignin becoming available with longer milling, although the trend is not observed for other fractions as described later on.

**Table 1:** ^13^C-IS Py-GC-MS Relative Abundance of Lignin-Derived Compounds in N24 Birch Wood and Fractions Resulting from Extraction 1 (Figure 2), Corrected for Relative Response Factors and Relative Abundance of ^13^C Analogues^a^

	fraction						
	ball-milled wood	WWE	WWE residue	alkaline	alkaline residue	IL/EtOH, H^+^	IL/EtOH, H^+^ residue
Lignin Subunits (%)							
H	1.3 ± 0.0	1.5 ± 0.0	2.6 ± 0.3	0.7 ± 0.0	2.9 ± 0.0	0.9 ± 0.0	6.6 ± 0.7
G	23.6 ± 0.1	18.0 ± 0.0	25.3 ± 1.2	26.3 ± 0.0	25.6 ± 0.0	33.0 ± 0.2	19.3 ± 1.3
S	75.1 ± 0.2	80.6 ± 0.0	72.1 ± 1.5	73.1 ± 0.0	71.6 ± 0.1	66.1 ± 0.2	74.1 ± 2.0
S/G	3.2 ± 0.0	4.5 ± 0.0	2.9 ± 0.0	2.8 ± 0.0	2.8 ± 0.0	2.0 ± 0.0	3.9 ± 0.4
*t*-CouA^b^	0.2 ± 0.1	0.2 ± 0.0	0.3 ± 0.0	0.3 ± 0.0	0.3 ± 0.0	0.3 ± 0.0	0.2 ± 0.1
*t*-ConA^c^	22.4 ± 0.2	16.5 ± 0.1	22.4 ± 0.4	23.3 ± 0.0	23.9 ± 0.0	30.8 ± 0.3	16.3 ± 1.4
*t*-SinA^d^	77.4 ± 0.2	83.4 ± 0.1	77.3 ± 0.4	76.5 ± 0.0	75.9 ± 0.0	69.0 ± 0.3	83.5 ± 1.4
*t*-SinA/*t*-ConA	3.5 ± 0.0	5.1 ± 0.1	3.4 ± 0.1	3.3 ± 0.0	3.2 ± 0.0	2.2 ± 0.0	5.1 ± 0.5
Structural Moieties (%)							
unsubstituted	4.9 ± 0.4	4.6 ± 0.0	7.1 ± 0.6	4.0 ± 0.0	11.3 ± 0.0	4.7 ± 0.1	9.5 ± 1.3
methyl	2.2 ± 0.3	1.4 ± 0.0	4.2 ± 0.5	2.5 ± 0.1	4.6 ± 0.0	2.1 ± 0.0	3.8 ± 0.0
vinyl	13.1 ± 1.2	10.3 ± 0.1	17.9 ± 0.6	11.5 ± 0.1	24.0 ± 0.1	10.3 ± 0.1	14.1 ± 1.1
C_α_-ox	5.7 ± 0.3	8.9 ± 0.0	7.4 ± 0.4	5.8 ± 0.1	4.1 ± 0.1	4.9 ± 0.2	7.9 ± 0.2
diketones	0.6 ± 0.0	1.1 ± 0.0	0.8 ± 0.0	0.5 ± 0.0	0.3 ± 0.0	0.6 ± 0.0	1.4 ± 0.1
C_β_-ox^e^	1.5 ± 0.1	1.9 ± 0.0	2.3 ± 0.1	1.3 ± 0.0	1.8 ± 0.0	1.6 ± 0.0	4.3 ± 0.0
Cγ-ox	66.7 ± 0.2	69.0 ± 0.0	49.6 ± 2.5	69.7 ± 0.3	39.9 ± 0.1	71.7 ± 0.3	52.9 ± 2.6
miscellaneous	5.9 ± 0.3	3.9 ± 0.1	11.6 ± 0.3	5.3 ± 0.0	5.3 ± 0.0	4.6 ± 0.1	7.5 ± 0.5
PhCγ^f^	74.5 ± 0.2	75.8 ± 0.1	63.6 ± 2.2	76.5 ± 0.3	55.6 ± 0.1	78.1 ± 0.2	65.3 ± 2.2
PhC_γ_-corrected^g^	73.9 ± 0.2	74.7 ± 0.1	62.9 ± 2.2	76.0 ± 0.3	55.3 ± 0.1	77.6 ± 0.2	63.9 ± 2.2

a Sum on the basis of structural classification in Table S1. Average and standard deviation of analytical duplicates.

b *trans*-Coumaryl alcohol.

c *trans*-Coniferyl alcohol.

d *trans*-Sinapyl alcohol.

e Excluding diketones.

f Phenols with intact α, β, γ carbon side chain.

g Phenols with intact α, β, γ carbon side chain, excluding diketones.

**Table 2:** Semi-Quantitative ^1^H–^13^C HSQC NMR Structural Characterization of Fractions from N24 Birch Following Extraction 1 (Figure 2)

	WWE	alkaline	IL/EtOH, H^+^
Lignin Subunits (%)^a^			
H	0.0	0.0	0.0
G	12.2	23.4	28.2
G_ox_	0.0	1.5	2.7
S	63.5	59.4	52.8
S_ox_	12.2	7.9	8.2
S/G	7.2	3.0	2.2
Interunit Linkages (per 100 C9 units)^b^			
β-O-4 aryl ether	43.3	57.8	28.1
β-O-4 aryl ether Cα-etherified	0.0	0.0	29.8
β-5 phenylcoumaran	0.0	2.6	3.2
β–β resinol	3.3	7.1	5.1
total	46.6	67.5	66.2
End-Units (per 100 C9 units)^b^			
cinnamyl alcohol		traces	0.4

a Relative distribution of lignin subunits (H + G + G_ox_ + S + S_ox_ = 100).

b Relative volume integral of substructure versus volume integral of total lignin subunits.

Py-GC-MS allowed the analysis of the insoluble residues obtained after each extraction step for extraction 1 (Figure 2), which enabled us to monitor the structural changes occurring at each step individually as well as for the extraction scheme as a whole. In general, the insights provided by Py-GC-MS and HSQC NMR (Table 2) on the soluble extracts were well aligned in terms of subunit composition, linkage content, and abundance of oxidized moieties.

Py-GC-MS analysis revealed a selective extraction of S-units into the WWE (S/G 4.5), as compared to the ball-milled birch wood (S/G 3.2) and warm water residue (S/G 2.9) (Table 1), as also observed by the HSQC NMR analysis of the WWE (S/G 7.2) (Table 2). This was evident both when considering overall H/G/S lignin-derived pyrolysis product distributions and the more specific 4-hydroxyphenylpropanoid distributions (coumaryl alcohol (tCouA)/coniferyl alcohol (tConA)/sinapyl alcohol (tSinA)). The WWE showed an S/G of 4.5 and t-SinA/t-ConA of 5.1, substantially higher than the ratios found in the residue at 2.9 and 3.4, respectively.

Previous work by Santos et al.^40^ reported S/G ratios of lignin from ten hardwood species, isolated as milled wood lignin and calculated from NMR spectra. The range of values reported (S/G 1.2–3.2) varied between species and exhibits the challenging nature of hardwood lignin subunit composition analysis. Further complicating a comparison with the literature, S/G ratios can be calculated using a variety of methods. A comparison between methods for poplar in previous work by Happs et al.^41^ showed a variation in the S/G values determined for the wood analyzed. Thus, the reported values depend on the extraction method as well as the wood species and the chosen analytical method for their calculation, an aspect that is not always described clearly in reports. By using diverse extraction protocols and complementary analytical techniques applied to the same initial biomass, we were able to draw attention to these discrepancies in our own observations.

Besides subunit composition, Py-GC-MS analysis provided insight into the abundance of oxidized moieties (Table 1). As compared to the ball-milled wood, the WWE and residue showed an increased abundance of Cα-oxidized products, confirming the occurrence of some oxidation reactions during the ball milling process. Nonetheless, this oxidation was not accompanied by severe lignin depolymerization, as evidenced from the abundance of PhCγ moieties, previously shown to correlate well with the abundance of intact interunit linkages.^42,43^ In line with the selective extraction of syringyl units into the water extract, the residue was slightly depleted in PhCγ moieties and thus in intact interunit linkages.

### Alkaline Extraction Shows No Selectivity toward S/G Moieties

Unlike the warm water extraction, Py-GC-MS analysis indicated that alkaline extraction did not show any selectivity toward specific lignin subunits (Table 1), with both the alkaline extract and residual solid material being similar to the WWE residue in terms of S/G and *t*-SinA/*t*-conA ratios. HSQC NMR analysis confirmed the subunit composition and abundance in the alkaline extract (Table 2). Regarding the values of the S/G ratios reported in Tables 1 and 2, different methods for the S/G ratio determination can give different values. HSQC NMR and Py-GC-MS measure different entities, i.e., specific C–H correlations and monomeric pyrolysis products released, respectively, which likely underlines the fact that they differ in the absolute sense. Nonetheless, it has previously been carefully validated that both methods strongly correlate, despite consistently observing that the S/G ratios determined by HSQC NMR slightly exceed those determined by Py-GC-MS.^42^ One of the potential reasons could be that in the semi-quantitative analysis of lignin with HSQC NMR, the end-units are differently quantified compared to “core” structures due to their different relaxation behavior, generally leading to an overestimation of the end-units.^44^ Given the abundance of Ca-oxidized syringyl moieties in the WWE fraction, presumably primarily benzoic acids and benzaldehydes, we expect these moieties to be overrepresented, which in turn results in an overestimated S/G ratio as well. Both methods clearly highlight the selective extraction of syringyl units in the WWE fraction.

Following an alkaline extraction, the β-O-4′ content of samples that had been milled under either atmosphere for 12 and 24 h (extraction 1) was approximately on the same level, with a slightly higher β–β′ content appearing in the N_2_-milled samples (Table S3). The similar S/G content varying from 2.52 to 3.01 for all samples shows no selectivity toward S- or G-moieties, which was also confirmed by Py-GC-MS (Table 1). The abundance of oxidized S-units increases in alkaline-extracted samples as the milling time increases in any atmosphere (extraction 1, Table S3), which is in line with the trend observed for the WWEs. Similar observations are made for the alkaline fractions of Extraction 3 (Table S3). In addition, there are some oxidized G-moieties in these fractions that are not visible in the WWEs. Quantification of the hydroxyl content of all extracts calculated by ^31^P NMR are presented in Table S4. From the comparison, there is no significant change that can be attributed to the different milling atmospheres.

The order in which the alkaline and IL/EtOH, H^+^ extraction steps were performed had an effect on the extraction yield of the alkaline treatment. When alkaline extraction was performed directly after warm water extraction (extractions 1 and 3, Figure 1), similarly high yields were achieved. On the other hand, there was a significant reduction in the yield of the alkaline fraction obtained from extraction 2, in which the alkaline extraction was performed on the wood residue after an IL/EtOH, H^+^ extraction (Figure 2). At the same time, the total extraction yields of all three protocols were similar, which suggested that the overall extractability of lignin from birch did not depend on the extraction step followed as much as expected. Interestingly, when the molecular weights of these alkaline fractions were analyzed, there was no significant difference (Table S5).

### The Yield of Extraction Using Ionic Liquid and Ethanol Is Affected by the Use of Acid

With the introduction of [amim]Cl, the wood residue swells due to the solubilization of cellulose fibers. As a result, the structure opens up, enabling the extraction of lignin with ethanol. The use of ethanol as an extraction solvent was chosen due to its ability to solubilize lignin and its compatibility with the general principles of green chemistry. In addition, ethanol can protect against lignin depolymerization and/or side reactions by nucleophilic addition to the α-position, forming an ethoxylated structure, as was observed in organosolv extraction of spruce.^45,46^ In this work, the use of hydrochloric acid (HCl) seems to be important for the effective extraction of lignin.

**Figure 5:**
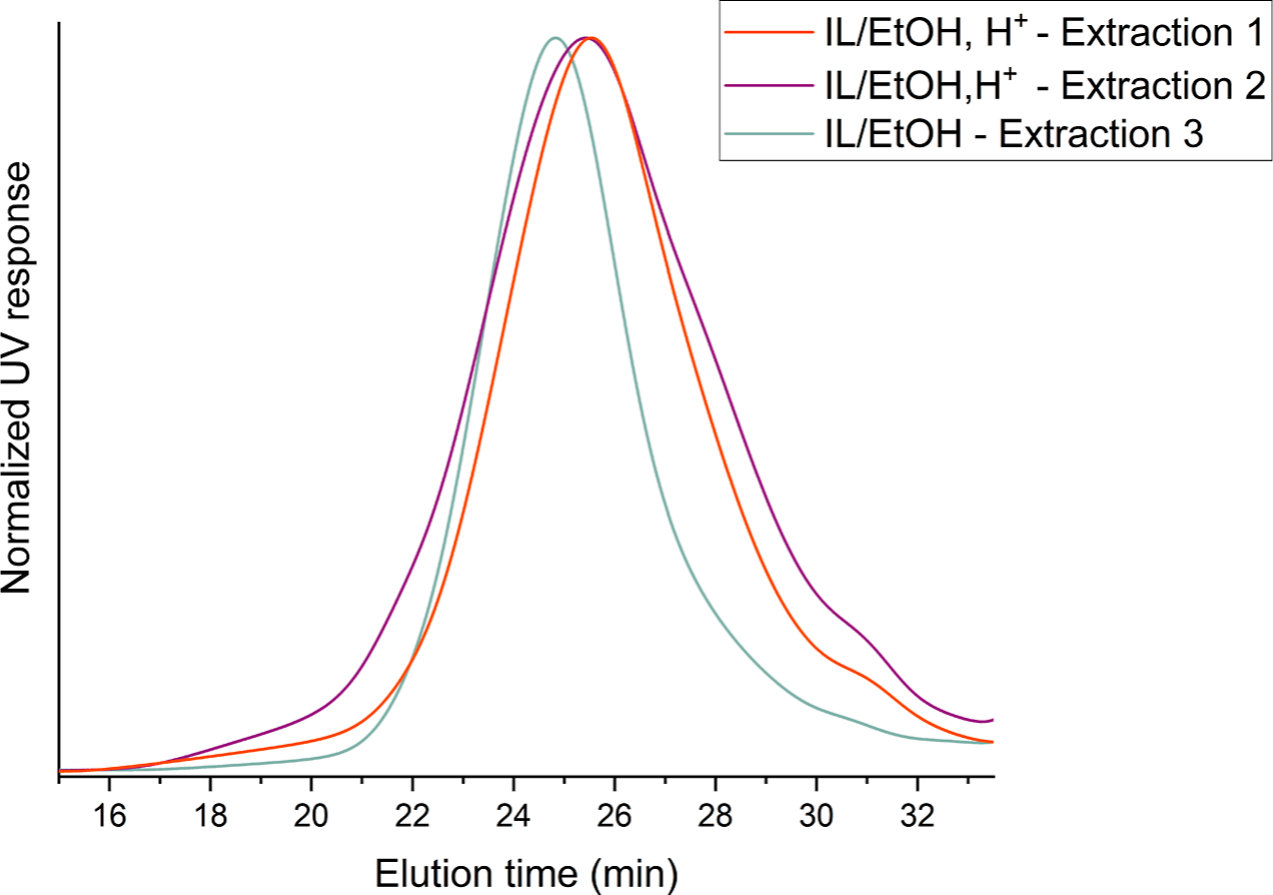
Size exclusion chromatogram overlays (THF–SEC) of A24 birch fractions obtained by IL/EtOH extraction, performed with or without HCl as indicated. The curves are normalized to a maximum value. Extraction methods are presented in Figure 2. The molecular weight values are reported in Table S5.

A comparison of extractions 1 and 3, which differ only in the use of acid in the final extraction step, shows that the yield is significantly lower in the second case, where no acid was used (Figure 2). In addition, the chromatogram overlay of these fractions (Figure 5) showed that the acid slightly affected the size of the lignin moieties extracted. In extraction 1, in which HCl was used, the molecular weight was lower and the dispersity of the sample slightly narrower compared to extraction 3. Py-GC-MS analysis confirmed that the G-units were selectively extracted during IL/EtOH, H^+^, when the extract was compared to the input (alkaline wood residue) and final wood residue following extraction 1 (Tables 1 and 2). Again, the final residue was found to be depleted of interunit linkages. A preferential extraction of lignin originating from the middle lamella is suggested in the literature for the early stages of extraction of milled wood lignin, which is known to be more condensed.^34^ However, the accumulation of condensed structures was seen at the end of our fractionation protocol, which would suggest the opposite. A retention of middle lamella lignin is not probable as with prolonged milling times, lignin form the secondary cell wall is also released.^11,34^ As a result, we attribute the accumulation of condensed structures in the residues to these lignin populations presumably being more recalcitrant to extraction, independent of the solvent system studied in this work. Oxidized moieties accumulated in the final residue, as in the previous residues, suggesting that there is ongoing (mild) oxidation throughout the extraction protocol.

Analysis of the interunit linkage composition of IL/EtOH, H^+^ fractions from extractions 1 and 2 (Table S3) shows that these were similar in terms of the most abundant linkages β–Ο-4′, β–β′, and β-5′, in both milling atmospheres, with only slight variations. In addition, the S/G ratio and the oxidized S- and G-units appeared to be similar for all fractions (Table S3). ^31^P NMR experiments showed a higher amount of guaiacyl –OH groups extracted in this step compared to that in the alkaline extraction. This was in accordance with HSQC NMR and Py-GC-MS, both showing a lower S/G ratio for the IL/EtOH, H^+^ extract compared to both the WWEs and alkaline extracts (Tables 1 and 2).

**Figure 6:**
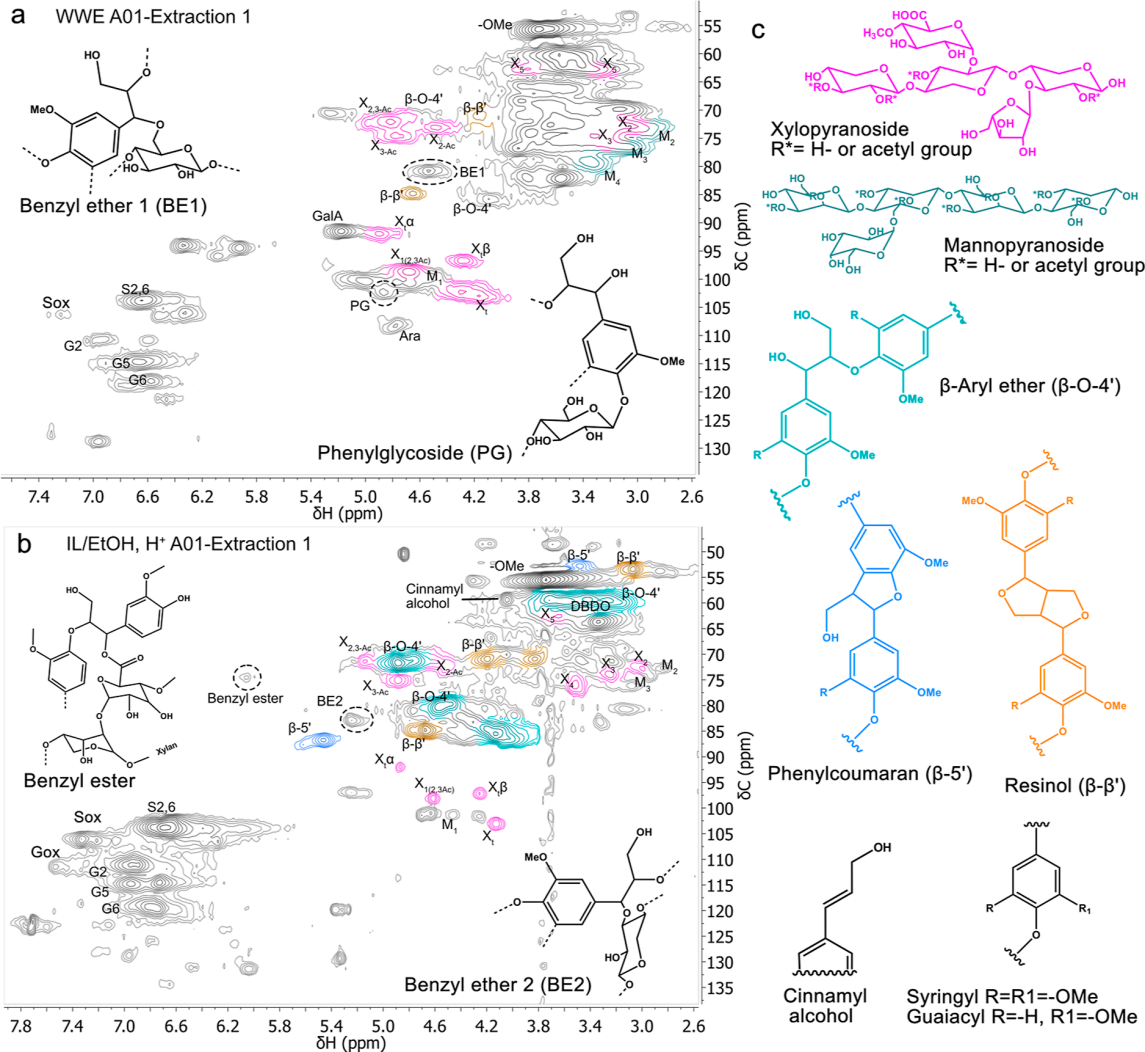
HSQC NMR spectra of (a) A01 WWE and (b) A01 IL/EtOH, H^+^ from extraction 1. The structures of the most abundant linkages are presented in (c).

In previous work, it was shown that hydrothermal pretreatment of wheat straw could cause lignin to relocate in the cell wall, in globular formations.^47^ Similar formations have been observed in softwoods after periodate pretreatment^48^ and in maize after dilute acid pretreatment.^21^ We hypothesized that similar changes could take place in our birch samples. In these previous works, lignin globular structures seemed to be on the surface of cellulose fibrils and are probably in contact with hemicelluloses, interacting through either hydrophobic interactions or covalent bonds (LCCs), both of which are affected by the pH. It was expected that protonated lignin with available hydroxyl groups at lower pH would be less likely to interact with the hydrophilic cellulose fibril surface, which could explain the higher extractability of lignin with the use of HCl in the IL/EtOH step. In addition, hydrophobic interactions have been shown to exist between acetylated xylan and lignin globules in dehydrogenative lignin experiments.^49^ These could be affected in the same way by low-pH conditions, enhancing lignin extraction. The alkaline conditions used in some extraction steps were not harsh enough to cleave all acetyl groups of native xylan, confirmed by the xylan acetyl group shifts in the NMR spectra of the alkaline extracts (Figure 6). In the case of covalent bonds, ether and ester LCCs could be cleaved and catalyzed by acid, thus increasing lignin extractability.

### LCCs in the Warm Water and IL/EtOH, H^+^ Extracts

NMR analysis of the IL/EtOH, H^+^ fractions from extraction 1 revealed the presence of a signal that we previously identified in WWEs of ball-milled spruce and attributed to a benzyl ester LCC (5.9/74.5 ppm).^11^ The presence of the bond in the birch samples is supported by the signals of carbohydrates in the C1 anomeric region (Figure 6b). The effect of ball milling on the abundance of this structure is obvious when comparing the intensities of the peak with the increasing milling time (Table S3). The same NMR shift was identified in the N_2_-milled samples, with the bond detected at 0.4% for the briefly milled samples and absent from the longer-milled samples. The higher-efficiency N_2_-milling (Figure 1) may explain why this bond disappeared faster in the N_2_-milled samples. Interestingly, the benzyl ester LCC appeared in the NMR spectra of samples that have been subjected to alkaline treatment, conditions that were expected to cause ester bonds to cleave. Nonetheless, these conditions indeed were found to be relatively mild given the fact that acetylated xylosyl moieties remained, as well. Conversely, it is possible that acid-catalyzed esterification occurred during the IL/EtOH, H^+^ extraction, which could lead to the formation of this LCC as an extraction artifact. However, the latter hypothesis could not explain the trend of reduced intensity of the benzyl ester bond with increasing milling time.

In addition to the benzyl ester, a benzyl ether between lignin and the C6/C5 of carbohydrates and a phenylglycoside structure were identified in the WWEs at 4.5/80.7 and 4.8/102.4 ppm, respectively. In the IL/EtOH, H^+^ extracts of extraction 1, a benzyl ether was identified at 5.2/83.0 ppm, which is formed between lignin and C2/C3/C4 of carbohydrates. The origin of the identified LCCs could be native and preserved in our extracts due to the use of mild conditions. However, there were indications of oxidation during the process that could lead to side reactions and the formation of these bonds during the extraction process.

## Outlook

Our data could be relevant for the development of green, high-yield protocols for the targeted extraction of lignin from biomass in lignin-first biorefinery processes and for high-end applications, where specific structural moieties are desired. As an example, the high S/G ratio of the WWE fraction makes it suitable for use in antioxidant materials. Likewise, a high LCC content could be advantageous in surfactant applications due to inherent amphiphilic properties, while those fractions with a higher hydroxyl content could be used in resins. These examples showcase that selecting appropriate extraction protocols that target desired lignin properties in the biomass is possible and could even eliminate the need for postextraction modifications.

We have demonstrated that the said structurally diverse lignin populations can be isolated using mild extraction technologies, i.e., by using green and environmentally benign solvents at low temperatures and low catalyst loadings. The dedicated valorization of these fractions now calls for further scrutinization of the energy and chemical consumption of the individual steps of the extraction protocol to determine the ultimate feasibility of derived biorefinery schemes in technoeconomic and carbon footprint terms.

## Conclusions

Despite major differences in the composition and structural features of hardwoods and softwoods, our results showed that the total extraction yield from hardwood birch was not markedly affected by the changes made to an extraction protocol initially developed for softwood spruce, although there were changes in the yields of the individual extracts. Comprehensive characterization of the fractions highlighted that warm water, alkali, and ionic liquid/ethanol solvent systems extracted fundamentally different lignin populations in terms of subunit composition, interunit linkage content, the abundance of oxidized moieties, and presence of ether- and ester-type LCCs. Taken together, these insights contribute to improving our understanding of the structure of (native) hardwood lignin and to its dedicated valorization.

## Supporting Information Available

NMR semi-quantitative determination of hemicelluloses, lignin, and lignin interunit linkages; DMSO–SEC overlays of WWEs; ^13^C-IS Py-GC-HR-MS of lignin-derived pyrolysis products; extraction yields ; HSQC NMR bond composition; hydroxyl content of extracts; and molecular weight determination of lignin-rich extracts with THF-SEC (PDF)
