# Supplementary material for: Impact of Extraction Method on the Structure of Lignin from Ball-Milled Hardwood
Source: ACS Sustain Chem Eng. 2023 Oct 19;11(43):15533–43. doi: 10.1021/acssuschemeng.3c02977 (PMC10618921; doi:10.1021/acssuschemeng.3c02977)
Supplement: Supplementary file 1 — sc3c02977_si_001.pdf [file sc3c02977_si_001.pdf]

Supporting Information for:

The impact of extraction method on the structure of lignin from ball milled hardwood

Ioanna Sapouna[a,b]*, Gijs van Erven[c,d], Emelie Heidling[b], Martin Lawoko[a,e]*, Lauren Sara McKee[a,b]

[a] Wallenberg Wood Science Center, KTH Royal Institute of Technology, Stockholm, Sweden

[b] Division of Glycoscience, Department of Chemistry, KTH Royal Institute of Technology, AlbaNova University Center, Stockholm, Sweden

[c] Wageningen Food and Biobased Research, Wageningen University & Research, Bornse Weilanden 9, 6708 WG, Wageningen, The Netherlands

[d] Laboratory of Food Chemistry, Wageningen University & Research, Bornse Weilanden 9, 6708 WG, Wageningen, The Netherlands

[e] Division of Wood Chemistry and Pulp Technology, Department of Fiber and Polymer Technology, KTH Royal Institute of Technology, Stockholm, Sweden

*To whom correspondence should be addressed at: Ioanna Sapouna sapouna@kth.se , Martin Lawoko lawoko@kth.se

Number of pages: 9

Number of tables: 5

Number of figures: 1

Supporting Information contents:

NMR calculations of relative hemicellulose and lignin content and lignin structural features

Figure S1: DMSO SEC overlays of normalized RI (dashed line) and UV responses (full line) of WWEs from Extraction 1. a, c, e, g are samples milled under normal atmosphere (A01-A24). b, d, f, h are samples milled under N_2 atmosphere (N01-N24).

Table S1: Identity and structural classification of lignin-derived pyrolysis products detected using ^{13}C-IS pyrolysis-GC-HR-MS.

Table S2: Average extraction yields from all protocols applied to ball milled birch. The error is the standard deviation of technical triplicates. Samples WWE A24 and WWE N24 were analyzed by NMR twice and the reported value is the average, with standard deviations ±0.5 and ±0.3 respectively. As a result of the small standard deviation, the % lignin in fraction for WWE A24 and WWE N24 in Extractions 2 and 3 was considered to be the same as Extraction 1 and are noted with one or two stars (*/**). It was not possible to obtain an NMR spectrum for the samples with a dash (-) due to low yield.

Table S3: Bond composition of the most characteristic lignin structures found in birch fractions, proportionally quantified using HSQC NMR. Total β-O-4' content was calculated by addition of the integrals for C_α/H_α in γ-hydroxylated β-O-4' (4.9/71.7 ppm), dibenzodioxocin (DBDO) (85.8/3.9 ppm) and, in the IL-fractions, the α-etherified β-O-4' (4.8-4.3/77-82 ppm). In WWE samples, β-O-4' content is calculated from the C_β/H_β in γ-hydroxylated β-O-4' (4.1/85.7 ppm). In spectra with overlapping signals it was not possible to get an accurate integration, so no value is reported (-). Assignments to benzyl ether 1 LCCs can heavily overlap with signals related to structures other than LCCs. As a result, interpretation of the reported values for this structure should be considered to be putative. Assignments were made according to the literature.[1, 2] Results are per 100 C9 units. Solvent used for analysis was DMSO-d_6.

Table S5: Molecular weight of alkaline and IL/EtOH extracts analyzed in THF-SEC after acetylation. Polystyrene standards were used for calibration. It is important to note that due to the different OH- content (**Table S4**) for the different fractions the degree of derivatization differs, which in turn affects the apparent molecular weight observed.

NMR calculations of relative hemicellulose and lignin content and lignin structural features

Estimations of the relative amounts of lignin and hemicelluloses in each fraction was performed using HSQC NMR spectra. First, the integral of C2/H2 in G-units and their oxidized structure, found at 6.9/110.4 ppm and 7.15/111.3 ppm respectively, was set to 100. Then the whole C1 anomeric area was integrated and the integral ($I_{anomeric}$) was divided by (100+ $I_{anomeric}$). This value corresponds to the relative amount of hemicelluloses in the fraction. Considering that the fraction consists of lignin and hemicelluloses, the remaining percentage is the one of lignin in the fraction. It is important to note that HSQC is a semi-quantitative method, hence the values are not absolute but rather represent a trend for comparison between samples, showing a relative proportion of compounds within each sample.

To calculate the relative amount of interunit linkages per 100 C9 units, the following steps were followed. The total C9 units were calculated by adding the integral of C2/H2 in G-units and its oxidized structure, at 6.9/110.4 ppm and 7.15/111.3 ppm respectively and half the integral of C2,6/H2,6 of S-units and its oxidized structure at 6.71/103.8 ppm and 7.2/106.1 ppm respectively.

The integral of each bond (I_{bond}) was divided by the total C9 units and multiplied by 100.

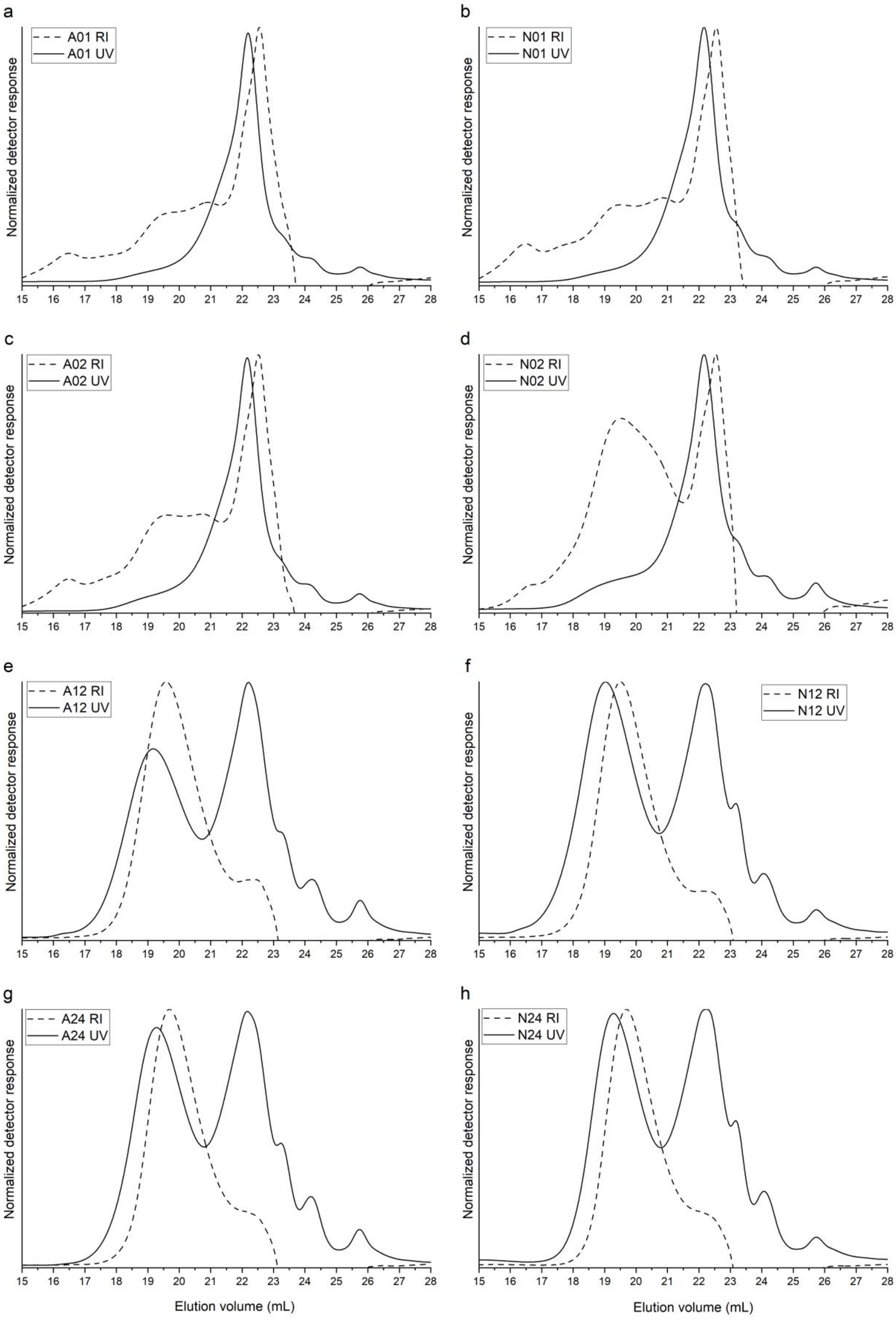

Figure S1: DMSO SEC overlays of normalized RI (dashed line) and UV responses (full line) of WWEs from Extraction 1. a, c, e, g are samples milled under normal atmosphere (A01-A24). b, d, f, h are samples milled under N_2 atmosphere (N01-N24).

Table S1: Identity and structural classification of lignin-derived pyrolysis products detected using ^{13}C-IS pyrolysis-GC-HR-MS.

#	Compound	CAS	Retention time (min)	Structural feature	Sidechain length	M_w ^{12}C (g·mol^{-1})	Quan ion ^{12}C [M-*e*]	M_w ^{13}C (g·mol^{-1})	Quan ion ^{13}C [M-*e*]
1	phenol	108952	9.61	H, unsub.	0	94	94.04132	100	100.06145
2	guaiacol	90051	9.91	G, unsub.	0	124	124.05188	131	115.04853
3	2-methylphenol	95487	10.87	H, methyl	C_α	108	108.05698	115	115.08045
4	4-methylphenol (+3-MP)	106445	11.83	H, methyl	C_α	108	107.04914	115	114.07263
5	4-methylguaiacol	93516	12.58	G, methyl	C_α	138	138.06753	146	146.09437
6	2,4-dimethylphenol	105679	13.06	H, methyl	C_α	122	107.04914	130	114.07263
7	4-ethylphenol	123079	14.12	H, misc.	C_β	122	107.04914	130	114.07263
8	4-ethylguaiacol	2785899	14.73	G, misc.	C_β	152	137.05971	161	145.08654
9	4-vinylguaiacol	7786610	16.20	G, vinyl	C_β	150	150.06753	159	159.09754
10	4-vinylphenol	2628173	16.36	H, vinyl	C_β	120	120.05697	128	128.08381
11	eugenol	97530	16.80	G, misc.	C_γ	164	164.08318	174	174.11673
12	4-propylguaiacol	2785877	16.90	G, misc.	C_γ	166	137.05971	175	145.08654
13	syringol	91101	17.52	S, unsub.	0	154	154.06245	162	162.08928
14	*cis*-isoeugenol	97541	18.18	G, misc.	C_γ	164	164.08318	174	174.11673
15	4-propenylphenol	539128	19.14	H, misc.	C_γ	134	133.06479	143	142.09498
16	*trans*-isoeugenol	97541	19.49	G, misc.	C_γ	164	164.08318	174	174.11673
17	4-methylsyringol	6638057	19.74	S, methyl	C_α	168	168.07810	177	177.10829
18	vanillin	121335	19.83	G, C_α-O	C_α	152	151.03897	160	159.06581
19	4-propyneguaiacol	-	20.23	G, misc.	C_γ	162	162.06753	172	172.10108
20	4-alleneguaiacol	-	20.47	G, misc.	C_γ	162	162.06753	172	172.10108
21	homovanillin	5603242	21.25	G, C_β-O	C_β	166	137.05971	175	145.08654
22	4-ethylsyringol	14059928	21.46	S, misc.	C_β	182	167.07022	192	176.10046
23	vanillic acid methyl ester	3943746	21.72	G, C_α-O	C_α	182	182.05736	191	191.08766
24	acetovanillone	498022	21.73	G, C_α-O	C_β	166	151.03897	175	159.06581
25	4-hydroxybenzaldehyde	123080	22.52	H, C_α-O	C_α	122	121.02848	129	128.05189
26	4-vinylsyringol	28343228	22.80	S, vinyl	C_β	180	180.07810	190	190.11164
27	guaiacylacetone	2503460	22.92	G, C_β-O	C_γ	180	137.05971	190	145.08654
28	4-allylsyringol	6627889	23.22	S, misc.	C_γ	194	194.09373	205	205.13065
29	propiovanillone	1835149	23.67	S, C_α-O	C_γ	180	151.03897	190	159.06581
30	guaiacyl vinyl ketone	-	23.94	G, C_α-O	C_γ	178	151.03897	188	159.06581
31	guaiacyl diketone	2034608	24.14	G, C_α-O, C_β-O	C_γ	194	151.03897	204	159.06581
32	cis-4-propenylsyringol	26624135	24.36	S, misc.	C_γ	194	194.09373	205	205.13065
33	4-propynesyringol	-	25.03	S, misc.	C_γ	192	192.07810	203	203.11500
34	4-allenesyringol	-	25.24	S, misc.	C_γ	192	192.07810	203	203.11500
35	trans-4-propenylsyringol	26624135	25.71	S, misc.	C_γ	194	194.09373	205	205.13065
36	dihydroconiferyl alcohol	2305137	25.72	S, C_γ-O	C_γ	182	137.05971	192	145.08654
37	syringaldehyde	134963	26.15	S, C_α-O	C_α	182	182.05736	191	191.08755
38	cis-coniferyl alcohol	458355	26.30	G, C_γ-O	C_γ	180	137.05971	190	145.08654
39	cis-coumaryl alcohol	3690059	26.50	H, C_γ-O	C_γ	150	107.04914	159	114.07263
40	homosyringaldehyde	-	27.13	S, C_β-O	C_β	196	167.07027	206	176.10046
41	acetosyringone	2478388	27.57	S, C_α-O	C_β	196	181.04954	206	190.07973
42	syringic acid methyl ester	884355	27.58	S, C_α-O	C_α	212	212.06793	222	222.10147
43	trans-coumaryl alcohol	3690059	27.61	H, C_γ-O	C_γ	150	107.04914	159	114.07263
44	trans-coniferyl alcohol	458355	28.04	G, C_γ-O	C_γ	180	137.05971	190	145.08654
45	trans-coniferaldehyde	458366	28.37	G, C_γ-O	C_γ	178	147.04406	188	156.07425
46	syringylacetone	19037582	28.47	S, C_β-O	C_γ	210	167.07027	221	176.10046
47	propiosyringone	5650431	29.16	S, C_α-O	C_γ	210	181.04954	221	190.07973
48	syringyl diketone	6925651	29.25	S, C_α-O, C_β-O	C_γ	224	181.04954	235	190.07973

49	syringyl vinyl ketone	-	29.43	S, C_α-O	C_γ	208	181.04954	219	190.07973
50	dihydrosinapyl alcohol	20736258	31.01	G, C_γ-O	C_γ	212	168.07841	223	177.10829
51	cis-sinapyl alcohol	537337	31.48	S, C_γ-O	C_γ	210	167.07027	221	176.10046
52	trans-sinapyl alcohol	537337	33.23	S, C_γ-O	C_γ	210	167.07027	221	176.10046
53	trans-sinapaldehyde	4206580	33.40	S, C_γ-O	C_γ	208	208.07301	219	219.10994

Table S2: Average extraction yields from all protocols applied to ball milled birch. The error is the standard deviation of technical triplicates. Samples WWE A24 and WWE N24 were analyzed by NMR twice and the reported value is the average, with standard deviations ±0.5 and ±0.3 respectively. As a result of the small standard deviation, the % lignin in fraction for WWE A24 and WWE N24 in Extractions 2 and 3 was considered to be the same as Extraction 1 and are noted with one or two stars (*/**). It was not possible to obtain an NMR spectrum for the samples with a dash (-) due to low yield.

Sample	Average extract (mg)	% lignin in fraction
**Extraction 1**		
WWE A01	15.80±0.26	13.7
WWE A02	18.60±2.65	-
WWE A12	120.07±4.03	11.1
WWE A24	198.77±4.54	11.0*
WWE N01	17.67±3.67	13.7
WWE N02	34.33±11.97	-
WWE N12	173.67±3.13	13.6
WWE N24	218.70±2.97	12.5**
ALKALINE A01	-	-
ALKALINE A02	-	-
ALKALINE A12	27.00±2.61	68.5
ALKALINE A24	78.20±15.24	58.1
ALKALINE N01	-	-
ALKALINE N02	-	-
ALKALINE N12	74.53±2.50	49.5
ALKALINE N24	119.27±5.94	66.5
IL/EtOH, H^+ A01	22.23±3.36	91.8
IL/EtOH, H^+ A02	52.50±12.13	92.2
IL/EtOH, H^+ A12	62.37±26.55	92.2
IL/EtOH, H^+ A24	50.23±5.83	97.8
IL/EtOH, H^+ N01	37.40±7.13	90.9
IL/EtOH, H^+ N02	44.23±9.34	91.2
IL/EtOH, H^+ N12	59.13±9.20	97.5
IL/EtOH, H^+ N24	33.47±2.19	98.4

**Extraction 2**		
WWE A24	199.20±6.40	*
WWE N24	212.40±2.02	**
IL/EtOH, H+ A24	82.90±4.81	95.7
IL/EtOH, H+ N24	77.70±7.01	96.9
ALKALINE A24	14.93±6.80	-
ALKALINE N24	9.63±4.75	-
**Extraction 3**		
WWE A24	195.97±1.36	*
WWE N24	221.77±2.12	**
ALKALINE A24	75.43±5.27	79.4
ALKALINE N24	110.23±2.50	76.8
IL/EtOH A24	17.20±0.60	-
IL/EtOH N24	15.03±3.98	-

Table S3: Bond composition of the most characteristic lignin structures found in birch fractions, proportionally quantified using HSQC NMR. Total β-O-4' content was calculated by addition of the integrals for C_α/H_α in γ-hydroxylated β-O-4' (4.9/71.7 ppm), dibenzodioxocin (DBDO) (85.8/3.9 ppm) and, in the IL-fractions, the α-etherified β-O-4' (4.8-4.3/77-82 ppm). In WWE samples, β-O-4' content is calculated from the C_β/H_β in γ-hydroxylated β-O-4' (4.1/85.7 ppm). In spectra with overlapping signals it was not possible to get an accurate integration, so no value is reported (-). Assignments to benzyl ether 1 LCCs can heavily overlap with signals related to structures other than LCCs. As a result, interpretation of the reported values for this structure should be considered to be putative. Assignments were made according to the literature.[1, 2] Results are per 100 C9 units. Solvent used for analysis was DMSO-d_6.

**Sample**	**% Total β-O-4'**			**% β-β'**	**% β-5'**	**S/G**	**Cα-oxidised S-units**	**Cα-oxidised G-units**	**Benzyl ether 1**	**Benzyl ether 2**	**Benzyl ester**
	β-O-4'	DBDO	α-etherified								
**Extraction 1**											
WWE A01	24.0	-	-	10.4	-	3.0	4.1	-	36.1	-	-

WWE A12	36.1	2.1	-	3.0	-	6.4	9.9	-	-	-	-
WWE A24	39.2	0.7	-	3.1	-	5.8	12.6	-	-	-	-
WWE N01	32.4	-	-	9.3	-	2.8	4.4	-	38.8		
WWE N12	44.2	-	-	4.6	-	7.2	9.6	-	-	-	-
WWE N24	43.3	-	-	3.3	-	7.2	12.2	-	-	-	-
ALKALINE A12	62.2	-	-	6.4	2.7	2.5	7.7	2.0	-	1.6	-
ALKALINE A24	56.0	-	-	6.4	2.4	2.7	9.7	2.6	-	1.8	-
ALKALINE N12	57.4	-	-	8.1	2.9	2.6	7.6	3.4	-	1.3	-
ALKALINE N24	57.8	-	-	7.1	2.6	3.0	7.9	1.5	-	1.9	-
IL/EtOH, H^+ A01	31.2	-	26.5	5.4	3.3	2.2	3.6	2.6	-	1.4	0.5
IL/EtOH, H^+ A02	29.7	-	28.8	5.3	3.1	2.1	4.5	2.7	-	1.4	0.3
IL/EtOH, H^+ A12	32.5	-	31.8	4.8	3.2	2.1	7.2	2.3	-	1.8	0.1
IL/EtOH, H^+ A24	26.1	-	29.0	5.9	3.3	2.0	9.3	2.9	-	2.3	-
IL/EtOH, H^+ N01	34.8	-	27.0	4.9	3.4	2.1	4.1	1.6	-	1.4	0.4
IL/EtOH, H^+ N02	23.4	-	30.3	5.5	3.6	2.2	6.5	2.8	-	1.7	0.4
IL/EtOH, H^+ N12	25.3	-	28.1	5.2	3.4	2.0	6.7	1.8	-	1.4	-
IL/EtOH, H^+ N24	28.1	-	29.8	5.1	3.2	2.2	8.2	2.7	-	1.6	-
Extraction 2											
IL/EtOH, H^+ A24	28.8	-	25.5	5.2	3.5	2.2	9.2	2.5	-	2.4	-
IL/EtOH, H^+ N24	29.0	-	31.3	5.5	3.9	2.0	7.8	3.3	-	1.7	-
Extraction 3											
ALKALINE A24	54.9	-	-	6.4	2.5	2.7	8.0	3.0	-	1.7	-
ALKALINE N24	56.7	-	-	6.4	2.7	2.8	7.2	2.2	-	1.2	-

Table S4: Hydroxyl content of extracts determined with quantitative ^{31}P-NMR. The values reported are the average of technical triplicates and the error is the standard deviation. The syringyl -OH signal (142.7 ppm) highly overlaps with the signals of other C_5-substituted structures (β-5', 4-O-5' and 5-5') in the region 140.2-143.0 ppm, so the integral cannot be used for quantification but is presented as a general trend. Note that contents are expressed in mmol/g sample and not in mmol/g lignin.

Sample	Aliphatic -OH (mmol/g)	C_5-substituted (mmol/g)	Guaiacyl -OH (mmol/g)	Syringyl -OH (mmol/g)	Carboxylic -OH (mmol/g)
**Extraction 1**					
ALKALINE A24	4.91 ± 0.37	0.45 ± 0.04	0.30 ± 0.02	0.05 ± 0.02	0.22 ± 0.02
ALKALINE N24	4.61 ± 1.18	0.50 ± 0.01	0.313 ± 0.003	0.08 ± 0.01	0.21 ± 0.01
IL/EtOH, H^+ A24	4.11 ± 0.07	0.59 ± 0.02	0.40 ± 0.01	0.07 ± 0.01	0.14 ± 0.01
IL/EtOH, H^+ N24	3.22 ± 0.64	0.56 ± 0.10	0.35 ± 0.06	0.04 ± 0.02	0.15 ± 0.02
**Extraction 2**					
IL/EtOH, H^+ A24	4.05 ± 0.06	0.57 ± 0.05	0.42 ± 0.02	0.06 ± 0.01	0.11 ± 0.06
IL/EtOH, H^+ N24	3.70 ± 0.05	0.71 ± 0.03	0.46 ± 0.01	0.095 ± 0.003	0.13 ± 0.01
**Extraction 3**					
ALKALINE A24	5.14 ± 0.03	0.46 ± 0.01	0.30 ± 0.01	0.062 ± 0.003	0.24 ± 0.01
ALKALINE N24	5.36 ± 0.16	0.53 ± 0.01	0.32 ± 0.01	0.08 ± 0.01	0.20 ± 0.02

Table S5: Molecular weight of alkaline and IL/EtOH extracts analyzed in THF-SEC after acetylation. Polystyrene standards were used for calibration. It is important to note that due to the different OH- content (**Table S4**) for the different fractions the degree of derivatization differs, which in turn affects the apparent molecular weight observed.

Sample	Mn (kDa)	Mw (kDa)	Đ
**Extraction 1**			
ALKALINE A12	3300	11000	3.23
ALKALINE A24	3900	12000	3.03
ALKALINE N12	2900	8600	2.96
ALKALINE N24	2600	8000	3.03
IL/EtOH, H^+ A01	2600	8200	3.14
IL/EtOH, H^+ A02	3100	11000	3.61
IL/EtOH, H^+ A12	2000	6800	3.48
IL/EtOH, H^+ A24	2600	8000	3.12
IL/EtOH, H^+ N01	2600	10000	4.04
IL/EtOH, H^+ N02	2300	9100	4.00
IL/EtOH, H^+ N12	2900	8500	2.97
IL/EtOH, H^+ N24	2000	6100	3.06
**Extraction 2**			
ALKALINE A24	4400	15800	3.63
ALKALINE N24	2800	10000	3.56

IL/EtOH, H^+ A24	2500	12700	4.97
IL/EtOH, H^+ N24	2300	7400	3.29
**Extraction 3**			
ALKALINE A24	3700	11100	3.04
ALKALINE N24	3500	12600	3.63
IL/EtOH A24	4900	10400	2.13
IL/EtOH N24	4300	9900	2.30

References

1. X. Meng, Y. Pu, C. G. Yoo, M. Li, G. Bali, D.-Y. Park, E. Gjersing, M. F. Davis, W. Muchero, G. A. Tuskan, T. J. Tschaplinski and A. J. Ragauskas, *ChemSusChem*, 2017, **10**, 139-150.
2. I. Sapouna and M. Lawoko, *Green Chemistry*, 2021, **23**, 3348-3364.
